# DNA Repair Gene XRCC1 Polymorphisms, Smoking, and Bladder Cancer Risk: A Meta-Analysis

**DOI:** 10.1371/journal.pone.0073448

**Published:** 2013-09-09

**Authors:** Shan Li, Qiliu Peng, Yongbin Chen, Jianpeng You, Zhiping Chen, Yan Deng, Xianjun Lao, Huiling Wu, Xue Qin, Zhiyu Zeng

**Affiliations:** 1 Department of Clinical Laboratory, First Affiliated Hospital of Guangxi Medical University, Nanning, Guangxi, People’s Republic of China; 2 Department of Traditional Chinese Medicine, First Affiliated Hospital of Guangxi Medical University, Nanning, Guangxi, People’s Republic of China; 3 Guangxi Medical University, Nanning, Guangxi, People’s Republic of China; 4 Department of Occupational Health and Environmental Health, School of Public Health at Guangxi Medical University, Nanning, Guangxi, People’s Republic of China; 5 Department of Geriatrics, First Affiliated Hospital of Guangxi Medical University, Nanning, Guangxi, People’s Republic of China; University of Hawaii Cancer Center, United States of America

## Abstract

**Background and Objective:**

The X-ray repair cross-complementing group 1 (XRCC1) protein plays a crucial role in base excision repair (BER) pathway by acting as a scaffold for other BER enzymes. Variants in the XRCC1 gene might alter protein structure or function or create alternatively spliced proteins which may influence BER efficiency and hence affect individual susceptibility to bladder cancer. Recent epidemiological studies have shown inconsistent associations between these polymorphisms and bladder cancer. To clarify the situation, a comprehensive meta-analysis of all available studies was performed in this study.

**Methods:**

PubMed, EMBASE, and Chinese Biomedical Literature database (CBM) databases have been systematically searched to identify all relevant studies for the period up to February 2013. Data were abstracted independently by two reviewers and Odds ratios (ORs) and 95% confidence intervals (CIs) were calculated. Subgroup analyses were performed mainly by ethnicity and smoking status.

**Results:**

A total of 26 case-control studies, including 24 studies for R399Q polymorphism, 15 studies for R194W polymorphism, and 7 studies for R280H polymorphism met the inclusion criteria and were selected. With respect to R399Q polymorphism, significantly decreased bladder cancer risk was found among smokers (AA vs. GG: OR=0.693, 95%CI= 0.515-0.932, *P*=0.015 and recessive model AA vs. GA+GG: OR=0.680, 95%CI= 0.515-0.898, *P*=0.007, respectively). With respect to R194W and R280H polymorphism, significantly increased bladder cancer risk were observed among Asians (TT+CT vs. CC:OR = 1.327, 95% CI 1.086-1.622, *P*=0.006 for R194W, and AA+GA vs. GG: OR=2.094, 95% CI 1.211–3.621, *P*=0.008 for R280H, respectively).

**Conclusions:**

This meta-analysis suggests that the XRCC1 R399Q polymorphism may play a protective role against bladder cancer among smokers. However, the XRCC1 R194W and R280H polymorphisms were both associated with increased bladder cancer risk among Asians. Further studies with larger sample sizes are needed to validate our finds.

## Introduction

Bladder cancer is one of the most common cancers of the urinary tract and a major problem worldwide [1]. The main known risk factors for bladder cancer include cigarette smoking, exposure to industrially related aromatic amines, and intake of drugs such as phenacetin, chlornaphrazine, and cyclophosphamide [2,3]. These exposures lead to DNA damage which, if remained unrepaired, may result in unregulated cell growth and even cancer [4]. DNA damage repair and cell cycle checkpoints facilitate cellular responses to DNA damage from endogenous and exogenous mutagenic exposures to maintain genomic integrity [5]. The base excision repair (BER) pathway is one of the four major DNA repair pathways in human cells. The proteins in the BER pathway mainly work on damaged DNA bases arising from endogenous oxidative and hydrolytic decay of DNA. Base damage and DNA single-strand breaks are mainly repaired through the BER pathway [6].

X-ray repair cross-complementing group 1 (XRCC1) is an essential DNA repair protein involved in BER pathway. The XRCC1 protein has no known catalytic activity but serves to orchestrate base excision repair via its role as a central scaffolding protein physically associated with DNA ligase III at its COOH terminus, DNA polymerase at its NH_2_ terminus, human AP endonuclease, polynucleotide kinase, and poly (ADP-ribose) polymerase, and via its function in recognizing and binding to single strand breaks [7–9]. Therefore, polymorphisms causing amino acid substitutions may impair the interaction of XRCC1 with the other enzymatic proteins and hence alter base excision repair activity.

Human XRCC1 gene maps to chromosome 19q13, 2 and is composed of 17 exons. It spans approximately 31.9kb, and encodes a protein of 633 amino acids. More than 300 validated single nucleotide polymorphisms (SNPs) in XRCC1 are listed in the dbSNP database, of which, approximately 35 variants are located in exons or promoter regions. The most extensively studied SNPs are R399Q on exon 10 (rs25487 in dbSNP, base 28152 G to A, Arg to Gln), R194W on exon 6 (rs1799782 in dbSNP, base 26304 C to T, Arg to Trp), and R280H on exon 9 (rs25489 in dbSNP, base 27466 G to A, Arg to His). These nonconservative amino acid alterations might influence DNA repair capability by altering the protein–protein interactions between XRCC1 and other BER proteins. Hence, it is biologically reasonable to hypothesize a potential relationship between XRCC1 polymorphisms and Bladder cancer risk. A study published in 2001 showed there was a protective effect for subjects that carried at least one copy of the codon 194 variant allele compared with those homozygous for the common allele (OR=0.59, 95% CI= 0.3–1.0) [10]. Subsequently, many studies have been published on this controversial issue, but it remains unclear whether there are significant associations between XRCC1 polymorphisms and bladder cancer risk. Small genetic association studies have various designs, different methodology, and insufficient power, and could inevitably increase the risk that chance could be responsible for their conclusions, while combining data from all eligible studies by meta-analysis has the advantage of reducing random error and obtaining precise estimates for some potential genetic associations. Therefore, we performed a meta-analysis of all available studies to clarify the effects of XRCC1 polymorphisms on bladder cancer risk.

## Materials and Methods

### Search strategy

This study was performed according to the proposal of Meta-analysis of Observational Studies in Epidemiology group (MOOSE) [11]. We conducted a comprehensive literature search in PubMed, Embase, and Chinese Biomedical Literature database (CBM) databases (up to February 15, 2013) using the following search strategy: (“Bladder cancer”) and (“X-ray repair cross-complementing group 1”, “XRCC1”, or “BER”) and (“polymorphism”, “variation”, “mutation”, “genotype”, or “genetic polymorphism”). There was no restriction on time period, sample size, population, language, or type of report. All eligible studies were retrieved and their references were checked for other relevant studies. The literature retrieval was performed in duplication by two independent reviewers (Shan Li and Qiliu Peng). When multiple publications reported on the same or overlapping data, we chose the most recent or largest population. When a study reported the results on different subpopulations, we treated it as separate studies in the meta-analysis.

### Selection criteria

Studies included in the meta-analysis were required to meet the following criteria: (1) Case–control studies which evaluated the association between XRCC1 polymorphisms and bladder cancer risk; (2) used an unrelated case–control design; (3) had an odds ratio (OR) with 95% confidence interval (CI) or other available data for estimating OR (95% CI); and (4) control population did not contain malignant tumor patients. Conference abstracts, case reports, editorials, review articles, and letters were excluded.

### Data extraction

Two separate investigators (Shan Li and Qiliu Peng) independently reviewed and extracted data from all eligible studies. Data extracted from eligible studies included the first author, year of publication, country of origin, ethnicity, genotyping method, matching criteria, source of control, bladder cancer confirmation, QC when genotyping, total numbers of cases and controls and genotype frequencies of cases and controls. Ethnic backgrounds were categorized as Caucasian, Asian, and African, and smoker status (smoker or nonsmoker) was additionally recorded for the stratified analysis. Smokers included current smokers and former smokers. Nonsmokers had never smoked. If a study did not state the ethnic descendent or if it was not possible to separate participants according to such phenotype, the group reported was termed as “mixed ethnicity”. To ensure the accuracy of the extracted information, the two investigators checked the data extraction results and reached consensus on all of the data extracted. If different results were generated, they would check the data again and have a discussion to come to an agreement. A third reviewer (Xue Qin) was invited to the discussion if disagreement still existed.

### Methodological quality assessment

Methodological quality was independently assessed by two reviewers (Shan Li and Qiliu Peng), according to a set of predefined criteria (Table 1) based on the scale of Thakkinstian et al. [12]. The revised criteria cover the credibility of controls, the representativeness of cases, assessment of bladder cancer, genotyping examination, Hardy-Weinberg equilibrium (HWE) in the control population, and association assessment. Disagreements were resolved by consensus. Scores ranged from 0 (lowest) to 12 (highest). Articles with scores less than 8 were considered ‘‘low-quality’’ studies, whereas those with scores equal to or higher than 8 were considered “high-quality’’ studies.

**Table 1:** Scale for Quality Assessment.

Criteria	Score
Representativeness of cases	
Selected from population or cancer registry	2
Selected from any urology /surgery service	1
Selected without clearly defined sampling frame or with extensive inclusion/exclusion criteria	0
Credibility of controls	
Population- or neighbor- based	3
Blood donors or volunteers	2
Hospital-based (cancer-free patients)	1
Healthy volunteers, but without total description	0.5
Urology patients	0.25
Not described	0
Ascertainment of bladder cancer	
Histological or pathological confirmation	2
Diagnosis of bladder cancer by patient medical record	1
Not described	0
Genotyping examination	
Genotyping done under ‘‘blinded’’ condition	1
Unblinded or not mentioned	0
Hardy-Weinberg equilibrium	
Hardy-Weinberg equilibrium in controls	2
Hardy-Weinberg disequilibrium in controls	1
No checking for Hardy-Weinberg disequilibrium	0
Association assessment	
Assess association between genotypes and bladder cancer with appropriate statistics and adjustment for confounders	2
Assess association between genotypes and bladder cancer with appropriate statistics without adjustment for confounders	1
Inappropriate statistics used	0

### Statistical analysis

The strength of the association between XRCC1 polymorphisms and bladder cancer risk was measured by odds ratios (ORs) with 95% confidence intervals (CIs). The significance of the pooled OR was determined by the Z test and a *p* value of less than 0.05 was considered significant. We assessed the associations of XRCC1 R399Q polymorphism with bladder cancer risk using additive genetic models (AA vs. GG and GA vs. GG), recessive genetic model (AA vs. GA+GG), and dominant genetic model (AA+GA vs. GG). However, with respect to R194W and R280H polymorphisms, the associations were assessed only by using dominant genetic model (TT+CT vs. CC for R194W, and AA+GA vs. GG for R280H, respectively) because of the low carrier rate of the mutate homozygote in the studied populations.

Two models of meta-analysis for dichotomous outcomes were conducted in this study: the random-effects model and the fixed-effects model. The random-effects model was conducted using the DerSimonian and Laird’s method [13], which assumed that studies were taken from populations with varying effect sizes and calculated the study weights both from in-study and between-study variances. The fixed-effects model was conducted using the Mantel–Haenszel’s method [14], which assumed that studies were sampled from populations with the same effect size and made an adjustment to the study weights according to the in-study variance. To assess the between-study heterogeneity, both the chi-square based *Q* statistic test to test for heterogeneity and the *I* ^2^ statistic to quantify the proportion of the total variation due to heterogeneity were calculated. Because of the low power of Cochran’s *Q* statistic, heterogeneity was considered significant when the results of the *Q* test was *P*_*Q*_ < 0.1 or *I* ^2^ ≥ 50%, and the random-effects model was used to pool the results. Otherwise, the fixed-effects model was used to pool the results when the result of the *Q* test was *P*_*Q*_ ≥ 0.1 and *I* ^2^ < 50%. Besides, the Galbraith plot was used to spot the outliers as the possible major sources of heterogeneity [15]. To better investigate possible sources of between-study heterogeneity, meta-regression analysis was also applied to both general analyses and subgroup analyses when heterogeneity was observed. To validate the credibility of outcomes in this meta-analysis, a sensitivity analysis was performed by sequential omission of individual studies or by omitting studies plotted by the Galbraith plot method as the possible major source of heterogeneity.

Subgroup analyses were performed by ethnicity, smoking status, and studies in HWE. Publication bias was investigated by funnel plot, in which the standard error of logor of each study was plotted against its logor. An asymmetric plot suggested possible publication bias. In addition, funnel-plot asymmetry was assessed by the method of Egger’s linear regression test [16]. The distribution of the genotypes in the control population was tested for HWE using a goodness-of-fit Chi-square test. All analyses were performed using Stata software, version 12.0 (Stata Corp., College Station, TX). All *p* values were two-sided. To ensure the reliability and the accuracy of the results, two authors imported the data into the statistic software program independently and got the same results.

## Results

### Study characteristics

**Figure 1:**
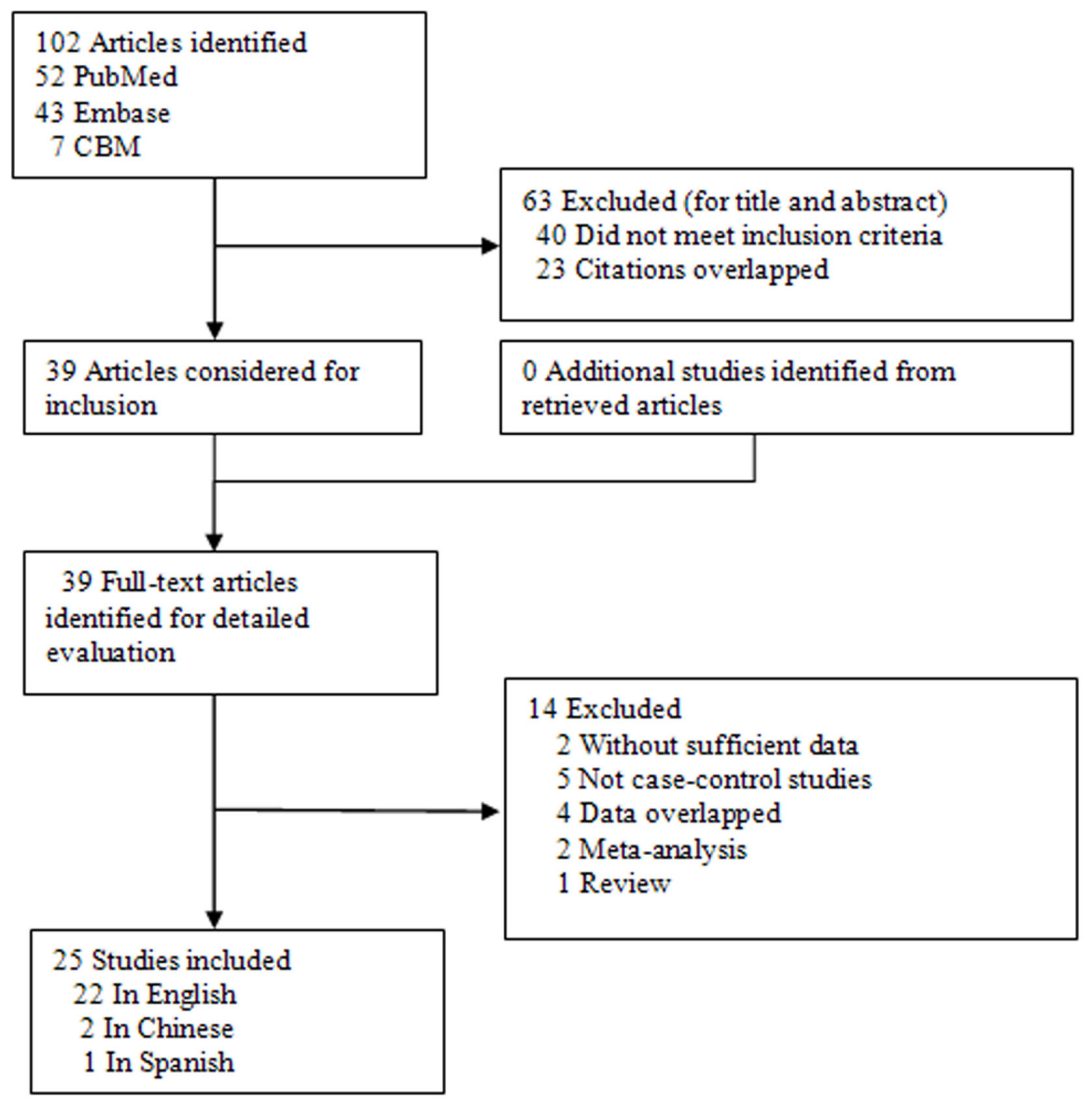
Flowchart of selection of studies for inclusion in meta-analysis.

With our search criteria, 102 individual records were found initially. After screening the titles and abstracts, 63 were excluded (40 did not examine XRCC1 R399Q, R194W and R280H Polymorphisms and bladder cancer risk, 23 were overlapped studies among the three databases) and only 39 full-text publications were preliminarily identified for further detailed evaluation (Figure 1). According to the exclusion criteria, 14 publications were excluded including 4 publications containing overlapping data [17–20], 2 for not presenting sufficient data for calculating OR and 95% CI [21,22], 5 were not case-control studies [23–27], 2 were meta-analysis [28,29] and one was a review [30]. Manual search of references cited in the eligible studies did not reveal any additional article As a result, a total of 25 relevant studies including 22 English articles [2,6,10,31–49], 2 Chinese papers (one was a dissertation of postgraduate student) [50,51], and one Spanish study [52] met the inclusion criteria for the meta-analysis. Among them, one of the eligible studies contained data on two different ethnic groups (African and Caucasian) [10], and we treated it independently. Therefore, a total of 26 separate comparisons were finally included in our meta-analysis. Among them, data were available from 24 individual case-control studies on R399Q polymorphism (including a total of 6750 bladder cancer cases and 8483 controls), 15 studies on R194W polymorphism (including a total of 5834 bladder cancer cases and 6492 controls), and 7 studies on R280H polymorphism (including a total of 2428 bladder cancer cases and 2442 controls). The main characteristics of the studies were presented in Table 2. Of all the eligible studies, 17 (including 6275 bladder cancer cases and 7702 controls) were conducted in Caucasian populations, 8 (including 1620 bladder cancer cases and 1853 controls) were in Asians, and 1 (including 19 bladder cancer cases and 13 controls) was in Africans. Seven studies (including 3173 bladder cancer cases and 4698 controls) were population–based and 18 (including 4109 bladder cancer cases and 4308 controls) were hospital–based studies. Sixteen articles (including 5947 bladder cancer cases and 7358 controls) of all eligible studies used quality control when genotyping and 6 (including 1613 bladder cancer cases and 1642 controls) studies in the present meta-analysis did not provide pathological or histological conformation for the bladder cancer diagnosis. Several genotyping methods were used, including PCR-RFLP, TaqMan assay, and MALDI-TOF. The genotype distributions of the controls in 2 studies were not consistent with HWE for R399Q polymorphism [32,45], 3 were not consistent with HWE for R280H polymorphism [10,39,49], and 1 was not consistent with HWE for R194W polymorphism [51].

**Table 2:** Characteristics of eligible studies.

First author (year)	Ethnicity (country)	Sample size (case/control)	Genotyping methods	BC confirmation	Source of control	Matching criteria	QC when genotyping	SNP studied	HWE(*P* value)			Quality scores
									R399Q	R280H	R194W	
Stern1 (2001)	Caucasian (America)	214/197	PCR-RFLP	HC	HB	Ethnicity, sex, and age	No	R399Q, R280H, R194W	0.923	**0.005**	0.185	7
Stern2 (2001)	African (America)	19/13	PCR-RFLP	HC	HB	Ethnicity, sex, and age	No	R399Q, R280H, R194W	0.512	**NA**	0.638	7
Shen (2003)	Caucasian (Italy)	201/214	PCR-RFLP	HC	HB	Age	No	R399Q	0.784	—	—	7.25
Sanyal (2004)	Caucasian (Sweden)	311/246	PCR-RFLP	NA	HB	Ethnicity, age, and region	Yes	R399Q	0.610	—	—	9
Kelsey (2004)	Caucasian (America)	355/544	PCR-RFLP	HC	PB	Age, sex, and region	Yes	R399Q	**0.031**	—	—	8.5
Matullo (2005)	Caucasian (Italy)	317/317	PCR-RFLP	HC	HB	Age, and region	Yes	R399Q, R194W	0.768	—	0.769	9
Broberg (2006)	Caucasian (Sweden)	61/155	MALDI-TOF	HC	PB	Ethnicity, age, and region	Yes	R399Q	0.840	—	—	10
Matullo (2006)	Caucasian (France et al.)	124/1094	TaqMan, Assay	PC	PB	Age, sex, and region	Yes	R399Q, R194W	0.632	—	0.171	9
Karahalil (2006)	Caucasian (Turkey)	146/100	PCR-RFLP	HC	HB	Age	No	R399Q	0.277	—	—	4
Wu (2006)	Caucasian (America)	696/629	TaqMan, Assay	HC	HB	Age, sex, and ethnicity	Yes	R399Q, R194W	0.339	—	0.317	6
Figueroa (2007)	Caucasian (Spain)	1150/1149	TaqMan, Assay	HC	PB	Age, sex, and region	Yes	R399Q, R280H, R194W	0.602	0.506	0.173	8
Sak (2007)	Caucasian (England)	532/562	TaqMan, Assay	NA	Mixed	Age, and sex	No	R399Q, R280H, R194W	0.953	**0.034**	0.450	9
Wu (2005)	Asian (China)	155/155	PCR-RFLP	HC	HB	Age, sex, and region	Yes	R399Q, R280H, R194W	0.616	0.167	0.060	9
Zhang (2006)	Asian (China)	242/225	PCR-RFLP	NA	PB	NA	Yes	R194W	—	—	**0.026**	10
Fontana (2008)	Caucasian (France)	51/45	TaqMan, Assay	HC	HB	NA	Yes	R399Q, R194W	0.264	—	0.693	6
Wang (2008)	Asian (China)	234/253	PCR-RFLP	HC	HB	Age, and sex	Yes	R399Q, R280H, R194W	0.065	0.068	0.069	9
Arizono (2008)	Asian (Janpan)	251/251	PCR-RFLP	NA	HB	Sex	No	R399Q	0.235	—	—	6
NARTER (2009)	Caucasian (Turkey)	83/45	PCR-RFLP	NA	HB	NA	No	R194W	—	—	0.352	5
Wen (2012)	Asian (China)	130/304	TaqMan, Assay	PC	HB	NA	No	R399Q	0.517	—	—	6.25
Zhi (2012)	Asian (China)	302/311	PCR-RFLP	PC	HB	NA	Yes	R399Q	0.059	—	—	8
Andrew (2008)	Caucasian (USA, Italy)	1029/1281	PCR-RFLP	HC	PB	Age, and sex	Yes	R399Q, R194W	**0.010**	—	0.094	10
Huang (2007)	Caucasian (USA)	614/600	TaqMan, Assay	HC	HB	Age, sex, and ethnicity	Yes	R399Q, R194W	NA*	—	NA*	8
Wen (2009)	Asian (China)	94/104	TaqMan, Assay	HC	HB	Age, sex, and region	Yes	R399Q	NA*	—	—	7.25
Covolo (2008)	Caucasian (Italy)	197/211	PCR-RFLP	HC	HB	Age, and region	No	R399Q	NA*	—	—	8
Gao (2010)	Caucasian (UK)	194/313	TaqMan, Assay	NA	HB	Age, and sex	No	R399Q	NA*	—	—	4
Mittal (2012)	Asian (India)	212/250	PCR-RFLP	HC	PB	Age, sex, and ethnicity	Yes	R399Q, R280H, R194W	0.276	**0.000**	0.985	8

HC, Histologically confirmed; PC, Pathologically confirmed; NA, Not available; QC, Quality control; PB, Population–based; HB, Hospital–based; HWE, Hardy–Weinberg equilibrium in control population; PCR–RFLP, Polymerase chain reaction-restriction fragment length polymorphism; MALDI-TOF, Matrix-assisted laser desorption/ ionization time-of-flight

NA*: The exact data of genotypes for calculating *P* value of HWE was not available, but were reported to be in HWE in the studies.

### Meta-analysis results

**Figure 2:**
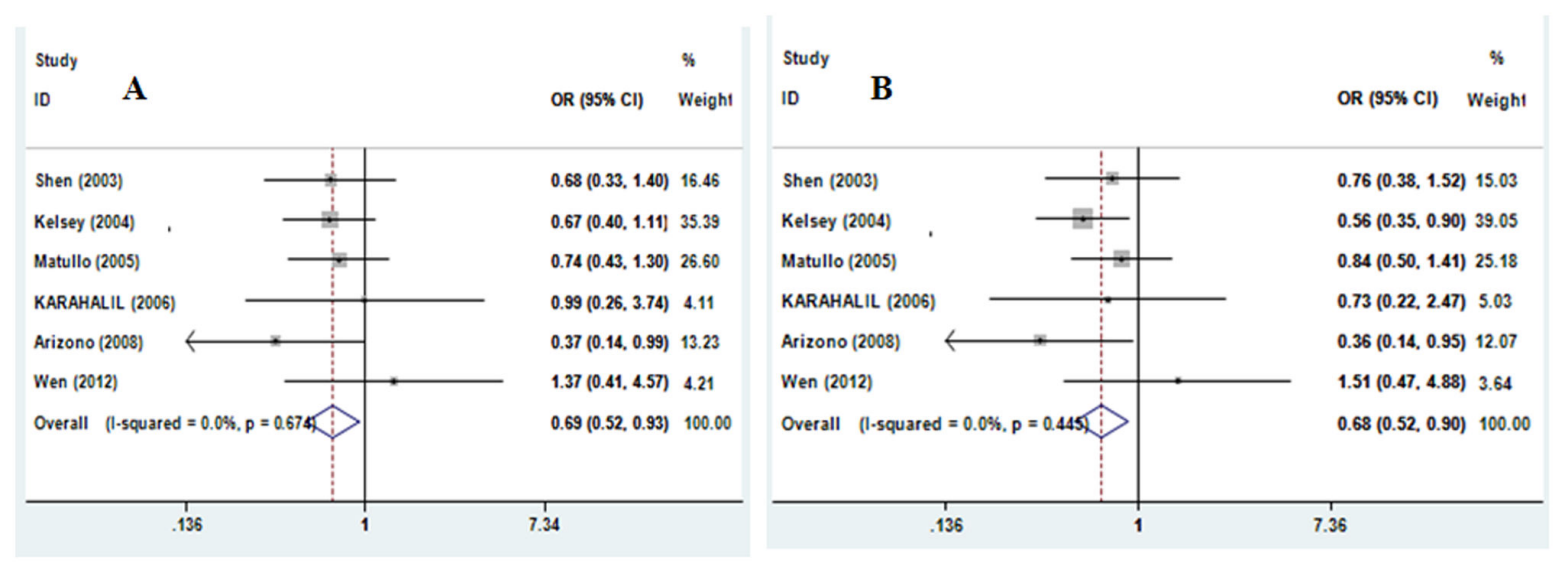
Forest plots of XRCC1 R399Q polymorphisms and bladder cancer risk among smokers. **A** Forest plots of XRCC1 R399Q polymorphism and bladder cancer risk among smokers using a fixed-effect model (contrast AA vs. GG); B Forest plots of XRCC1 R399Q polymorphism and bladder cancer risk among smokers using a Fixed-effect model (recessive model AA vs. GA+GG).

For the R399Q polymorphism, the between-study heterogeneity was significant when all studies were pooled into meta-analysis (*I* ^2^ =55.1%, *P*_*Q*_=0.002), thus, the random-effects model was used to pool the results. The results of pooling all studies showed that the R399Q polymorphism was not associated with bladder risk in all genetic models (additive models AA vs. GG and GA vs. GG, recessive model, and dominant model; Table 3). Moreover, we did not identified significant results between the R399Q polymorphism and bladder cancer risk in all comparison models in subgroup analyses according to ethnicity and the studies after excluding the subjects not in HWE. However, in the subgroup analysis stratified by smoking status, we found significantly decreased bladder cancer risk in genetic models AA vs. GG and recessive model AA vs. GA+GG (OR=0.693, 95%CI= 0.515-0.932, *P*=0.015 and OR=0.680, 95%CI= 0.515-0.898, *P*=0.007, respectively, Figure 2) in smokers, no significant association was found in all comparisons in non-smokers.

**Table 3:** Meta-analysis of the XRCC1 gene polymorphisms on bladder cancer risk.

Comparison	Population	No. of studies	Test of association			M	Test of heterogeneity		*P*_Egger_’_s test_
			OR	95% CI	*P* Value		*P*_*Q*_ Value	*I* ^2^ (%)	
R399Q									
AA vs. GG	Overall	19	0.884	0.733-1.066	0.195	R	0.002	55.1	0.202
	Caucasian	13	0.928	0.819-1.051	0.239	F	0.654	0.0	0.266
	Asian	6	0.762	0.376-1.544	0.450	R	0.000	83.6	0.085
	Smokers	6	**0.693**	**0.515-0.932**	**0.015**	F	0.674	0.0	0.670
	Non-smokers	7	1.060	0.723-1.555	0.765	F	0.816	0.0	0.667
	Studies in HWE	17	0.892	0.714-1.113	0.311	R	0.001	58.1	0.186
	Studies after excluding the outliers	17	0.934	0.831-1.049	0.249	F	0.469	0.0	0.268
GA vs. GG	Overall	20	1.064	0.989-1.145	0.096	F	0.090	31.4	0.721
	Caucasian	13	1.079	0.994-1.172	0.069	F	0.560	0.0	0.796
	Asian	6	0.965	0.727-1.280	0.804	R	0.010	66.8	0.176
	African	1	2.500	0.568-11.011	0.226	—	—	—	—
	Smokers	6	1.020	0.848-1.227	0.832	F	0.160	37.0	0.966
	Non-smokers	7	0.779	0.496-1.223	0.278	R	0.031	56.8	0.236
	Studies in HWE	18	1.032	0.950-1.122	0.458	F	0.134	27.6	0.907
	Studies after excluding the outliers	18	1.070	0.992-1.154	0.081	F	0.277	14.8	0.964
AA+GA vs. GG	Overall	24	1.006	0.922-1.097	0.892	R	0.036	37.1	0.365
	Caucasian	16	1.037	0.966-1.113	0.320	F	0.794	0.0	0.334
	Asian	7	0.908	0.674-1.221	0.552	R	0.001	74.8	0.130
	African	1	2.500	0.568-11.011	0.226	—	—	—	—
	Smokers	7	0.972	0.837-1.130	0.715	F	0.478	0.0	0.874
	Non-smokers	8	0.865	0.638-1.173	0.350	R	0.087	43.7	0.306
	Studies in HWE	22	0.988	0.896-1.091	0.815	R	0.030	39.7	0.408
	Studies after excluding the outliers	22	1.028	0.963-1.098	0.410	F	0.514	0.0	0.604
AA vs. GA+GG	Overall	19	0.867	0.736-1.023	0.091	R	0.010	48.5	0.238
	Caucasian	13	0.892	0.793-1.003	0.055	F	0.479	0.0	0.328
	Asian	6	0.782	0.433-1.412	0.415	R	0.000	78.7	0.169
	Smokers	6	**0.680**	**0.515-0.898**	**0.007**	F	0.445	0.0	0.738
	Non-smokers	7	1.088	0.758-1.561	0.648	F	0.830	0.0	0.826
	Studies in HWE	17	0.898	0.746-1.081	0.257	R	0.018	46.8	0.162
	Studies after excluding the outliers	17	0.899	0.805-1.003	0.058	F	0.414	3.5	0.338
R194W									
TT+CT vs. CC	Overall	15	1.008	0.909-1.118	0.880	F	0.247	18.5	0.166
	Caucasian	10	0.916	0.811-1.035	0.158	F	0.845	0.0	0.077
	Asian	4	**1.327**	**1.086-1.622**	**0.006**	F	0.848	0.0	0.121
	African	1	0.185	0.017-2.024	0.167	—	—	—	—
	Smokers	2	0.866	0.627-1.195	0.381	F	0.500	0.0	—
	Non-smokers	3	0.874	0.589-1.295	0.501	F	0.441	0.0	—
	Studies in HWE	14	0.983	0.882-1.095	0.754	F	0.333	11.1	0.152
R280H									
AA+GA vs. GG	Overall	7	**1.609**	**1.153-2.247**	**0.005**	R	0.002	70.7	0.507
	Caucasian	3	1.209	0.972-1.503	0.088	F	0.513	0.0	—
	Asian	3	**2.094**	**1.211-3.621**	**0.008**	R	0.006	80.2	—
	African	1	3.857	0.171-87.199	0.396	—	—	—	—

M, model; OR, odds ratio; CI, confidence intervals; R, random effects model; F, fixed effects model; HWE, Hardy–Weinberg equilibrium

**Figure 3:**
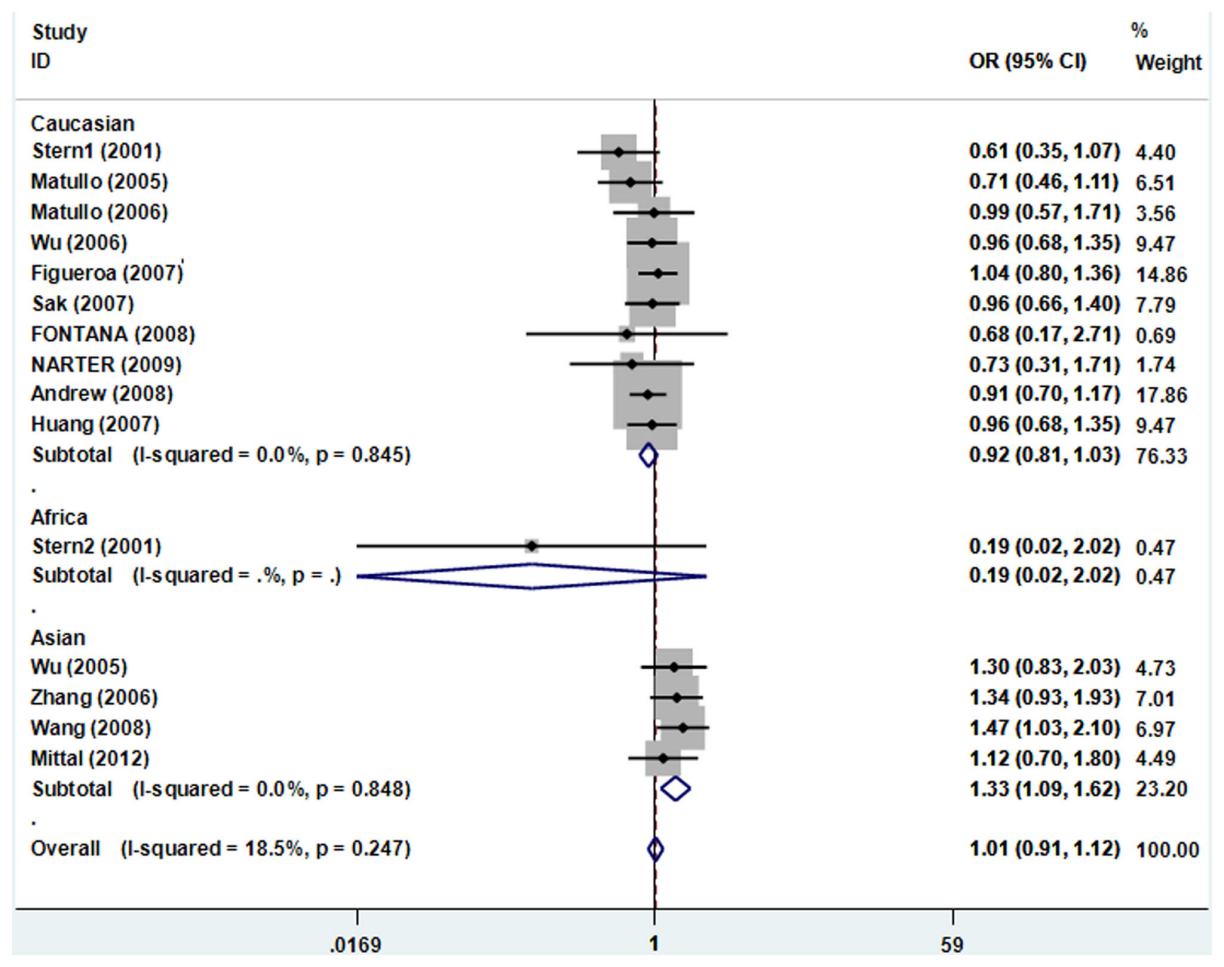
Forest plots of XRCC1 R194W polymorphisms and bladder cancer risk using a fixed-effect model (TT+CT vs. CC).

For the R194W polymorphism, there was no between-study heterogeneity when all 15 eligible studies were pooled into meta-analysis (*I* ^2^ = 18.5%, *P*_*Q*_ =0.247), thus the fixed-effects model was used to pool the results. The combined results showed that the R194W polymorphism was not associated with bladder cancer risk (Table 3). In the subgroup analyses by ethnicity, the results showed that the R194W polymorphism was associated with an increased bladder cancer risk among Asians (TT+CT vs. CC:OR = 1.327, 95% CI 1.086–1.622, *P*=0.006), while the association was also not found in Caucasians and Africans (Figure 3). Similarly, no any significant association was observed in subgroup analysis stratified by smoking status and the studies after excluding the subjects not in HWE.

**Figure 4:**
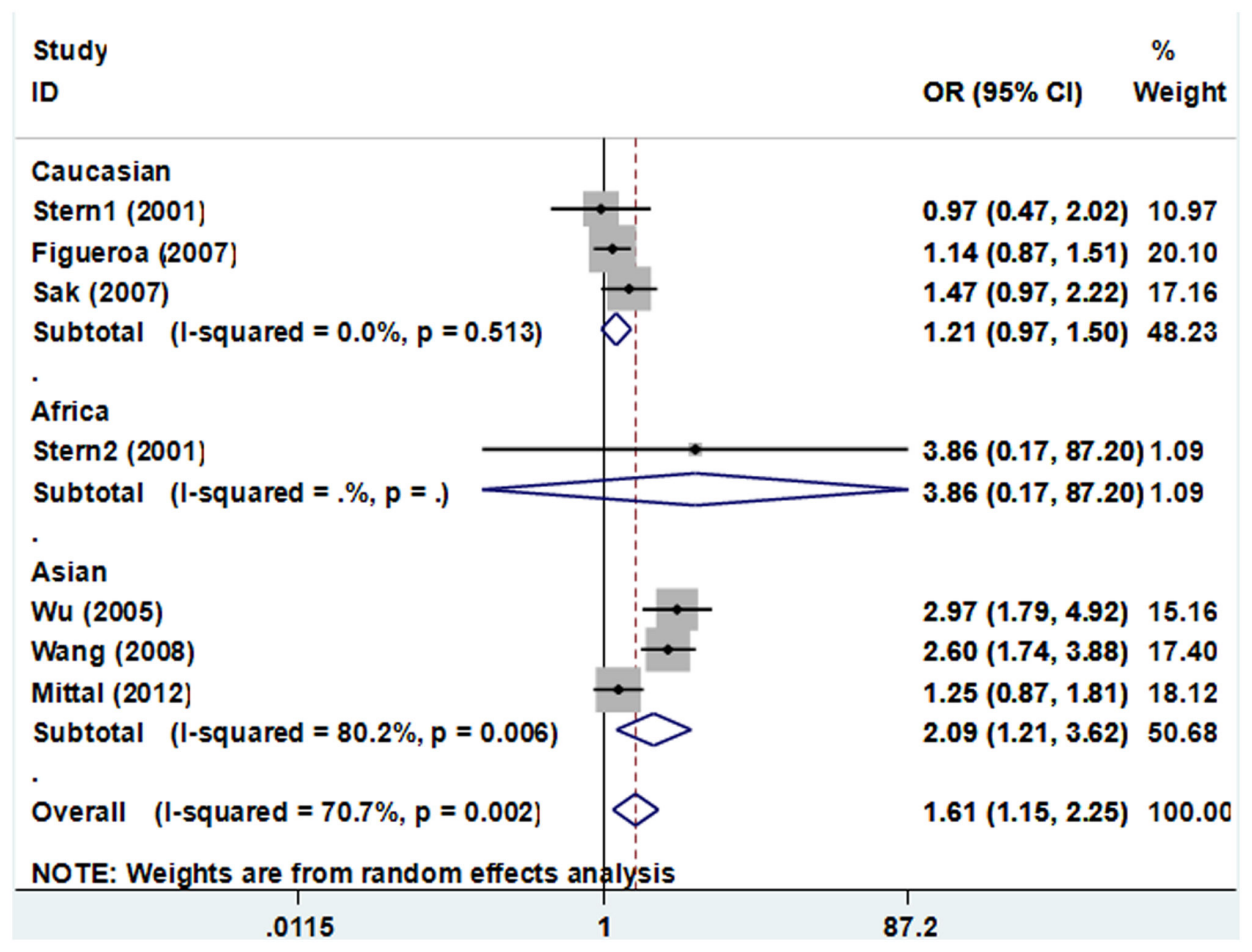
Forest plots of XRCC1 R280H polymorphisms and bladder cancer risk using a random-effect model (AA+GA vs. GG).

For the R280H polymorphism, obvious significant between-study heterogeneity was observed when all the eligible studies were pooled into meta-analysis (*I* ^2^ = 70.7%, *P*_*Q*_ = 0.002), thus the random-effects model was used to pool the results. The combined result showed that the R280H polymorphism was significantly associated with increased bladder cancer risk (AA+GA vs. GG: OR=1.609, 95% CI 1.153–2.247, *P*=0.005). In subgroup analyses by ethnicity, the results showed that the R280H polymorphism was associated with an increased bladder cancer risk among Asians (AA+GA vs. GG: OR=2.094, 95% CI 1.211–3.621, *P*=0.008, Figure 4).

### Heterogeneity analysis

**Figure 5:**
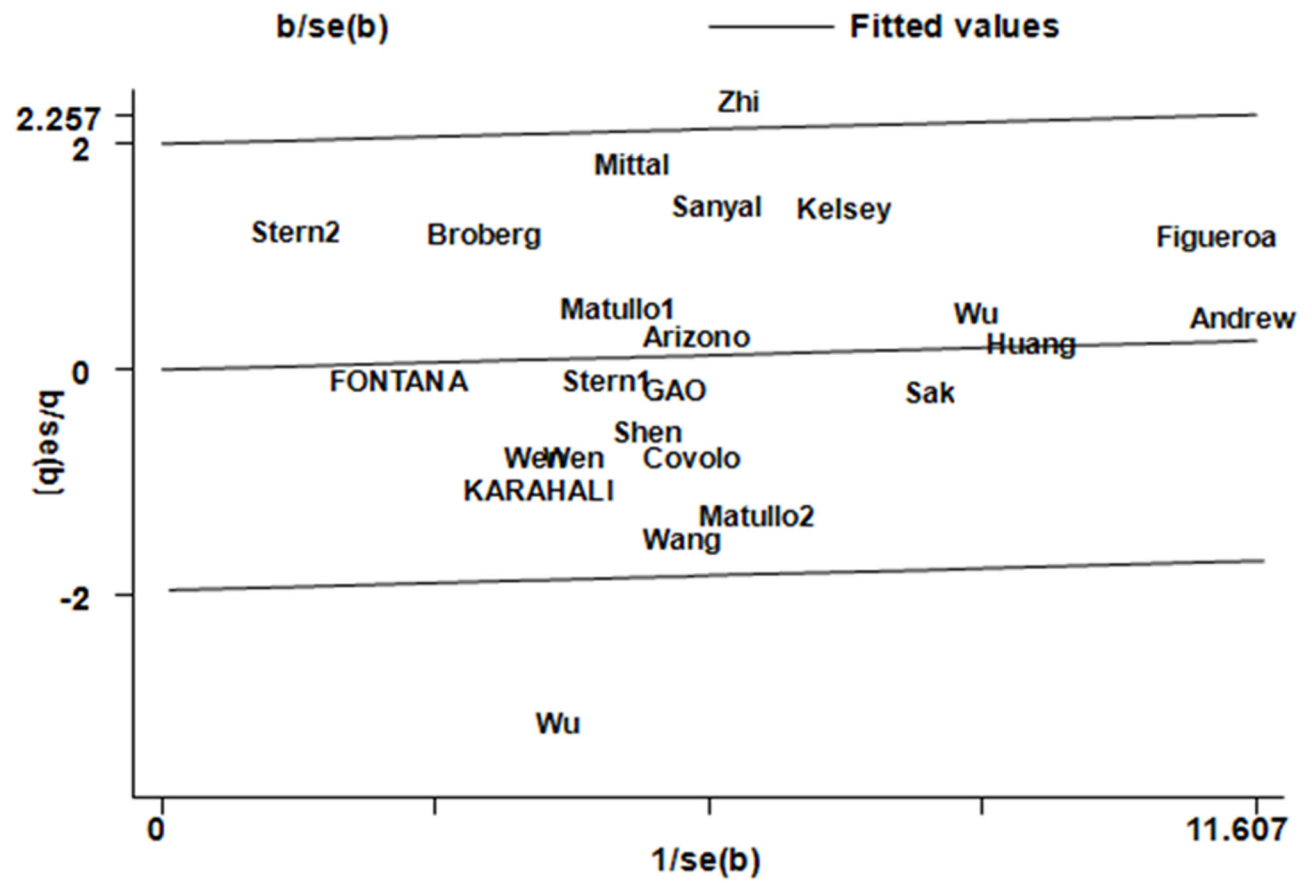
Galbraith plots of XRCC1 R399Q polymorphism and bladder cancer risk in dominant model AA+AG vs. GG. The studies of Wu et al. and Zhi et al. were spotted as outliers.

For the R399Q polymorphism, the *I* ^2^ values of heterogeneity were greater than 50% and the *P*_*Q*_ values were lower than 0.10 in additive model AA vs. GG, recessive model AA vs. GA+GG, and dominant model AA+GA vs. GG in the overall populations, which indicated statistically signiﬁcant heterogeneity among studies. To explore the sources of heterogeneity, we performed metaregression and subgroup analyses. Metaregression analysis of data showed that the ethnicity was the major source which contributed to heterogeneity. The Genotyping methods, Bladder cancer confirmation, Source of control, QC when genotyping, and Quality scores were not effect modifiers. Subsequently, we performed subgroup analyses stratified by ethnicity. However, heterogeneity still existed in all the above three genetic comparison models in Asians (Table 3). To further investigate the heterogeneity, we performed Galbraith plots analysis to identify the outliers which might contribute to the heterogeneity. Our results showed that the studies Wu et al. [50] and Zhi et al. [44] were outliers in additive model AA vs. GG, recessive model AA vs. GA+GG, and dominant model AA+GA vs. GG model for R399Q polymorphism (Figure 5). All *I* ^2^ values decreased obviously and *P*_*Q*_ values were greater than 0.10 after excluding the two studies Wu et al. [50] and Zhi et al. [44] in all genetic comparison models in the overall populations (additive model AA vs. GG: *P*_*Q*_ = 0.469, *I* ^2^ = 0.0; recessive model AA vs. GA+GG: *P*_*Q*_ = 0.414, *I* ^2^ = 3.5; dominant model AA+GA vs. GG: *P*_*Q*_ = 0.514, *I* ^2^ = 0.0), Asians (additive model AA vs. GG: *P*_*Q*_ = 0.107, *I* ^2^ = 46.8; recessive model AA vs. GA+GG: *P*_*Q*_ = 0.186, *I* ^2^ = 37.7; dominant model AA+GA vs. GG: *P*_*Q*_ = 0.101, *I* ^2^ = 48.5), and studies in HWE (additive model AA vs. GG: *P*_*Q*_ = 0.481, *I* ^2^ = 41.0; recessive model AA vs. GA+GG: *P*_*Q*_ = 0.670, *I* ^2^ = 0.0; dominant model AA+GA vs. GG: *P*_*Q*_ = 0.491, *I* ^2^ = 0.0). The signiﬁcance of the summary ORs for the R399Q polymorphism in different comparison models in the overall populations and subgroup analyses were not inﬂuenced by omitting the two studies.

**Figure 6:**
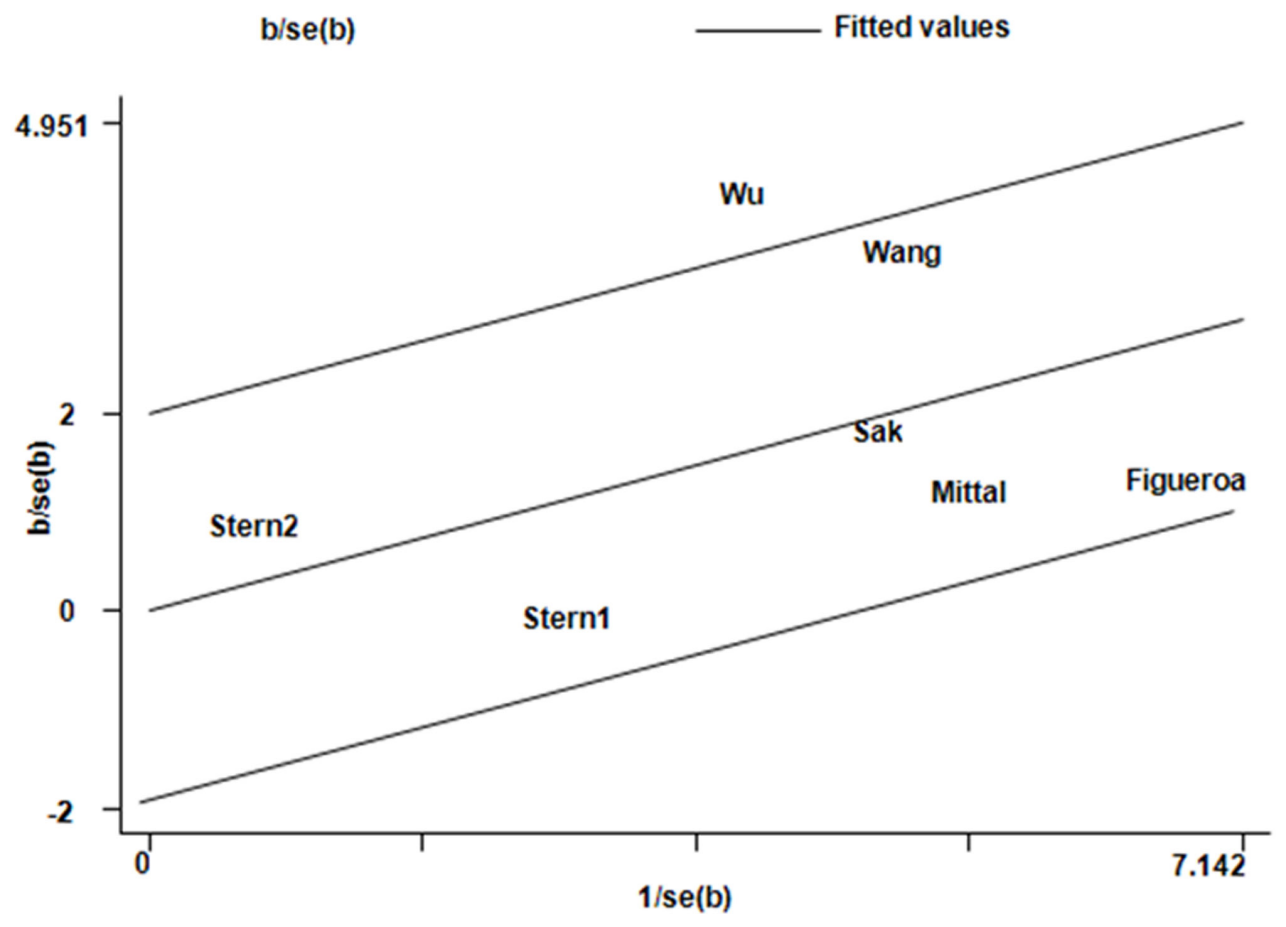
Galbraith plots of XRCC1 R280H polymorphism and bladder cancer risk in dominant model AA+GA vs. GG. The study of Wu et al. was spotted as outlier.

For the R280H polymorphism, significant between-study heterogeneity was also observed in the pooling analyses of total available studies (AA+GA vs. GG: *P*_*Q*_ = 0.002, *I* ^2^ = 70.7). Metaregression analysis of data showed that the Ethnicity, Genotyping methods, Bladder cancer confirmation, Source of control, QC when genotyping, and Quality scores were not effect modifiers. Galbraith plots analysis indicated that the study Wu et al. [50] was spotted as the major source of the heterogeneity (Figure 6). The *I* ^2^ values decreased obviously and *P*_*Q*_ values were greater than 0.10 after excluding the study Wu et al. [50] in the overall populations (AA+GA vs. GG: *P*_*Q*_ = 0.107, *I* ^2^ = 11.7) and Asians (AA+GA vs. GG: *P*_*Q*_ = 0.062, *I* ^2^ = 48.3). The signiﬁcance of the ORs for the R280H polymorphism in the overall population and subgroup analyses were not changed by omitting this study.

For the R194W polymorphism, we did not observe any significant between-study heterogeneity in the overall populations and the subgroup analyses.

### Sensitivity analysis

Sensitivity analysis was performed by sequential omission of individual studies. For analyses of pooling more than three individual studies, the significance of ORs was not influenced excessively by omitting any single study (data not shown). For the R399Q polymorphism, sensitivity analysis was further performed by omitting the studies by Kelsey et al. [32] and Andrew et al. [19], in which the control populations were not consistent with HWE, and the significance of all ORs were not altered after excluding these two studies (Table 3). For the R194W polymorphism, sensitivity analysis was also further performed by omitting the study by Zhang et al. [51] in which the control populations were significantly deviated from HWE, and the significance of all ORs was also not altered. For the R280H polymorphism, sensitivity analysis by omitting those studies whose control populations were deviated from HWE was not performed because it might be unacceptable and could cause some biases by excluding too many studies.

### Publication bias

Begg’s funnel plot and Egger’s test were performed to access the publication bias of literatures included in this meta-analysis. The shapes of Funnel plot did not reveal obvious evidence of asymmetry, and all the *p* values of Egger’s tests were more than 0.05, providing statistical evidence of the funnel plots’ symmetry. The results above suggested that publication bias was not evident in this meta-analysis.

## Discussion

Previous studies investigating the associations between XRCC1 Polymorphisms and bladder cancer risk have provided inconsistent results, and most of those studies involved no more than a few hundred bladder cancer cases, which is too few to assess any genetic effects reliably. Meta-analysis has been recognized as an important tool to more precisely define the effect of selected genetic polymorphisms on the risk for disease and to identify potentially important sources of between-study heterogeneity. A meta-analysis of 12 studies conducted in 2008 [28] showed that the XRCC1 R194W polymorphism might not be risk factors for bladder cancer, but the R399Q polymorphism associated with decreased susceptibility of bladder cancer under recessive model (OR=0.65, 95% CI= 0.49-0.86) and homozygote contrast (OR=0.66, 95% CI= 0.49-0.90) among ever smokers. Another meta-analysis [29], performed almost at the same time and quite similar in methods, showed there was no association between XRCC1 R399Q, R194W and R280H polymorphisms and bladder cancer susceptibility. The previous meta-analysis did not cover eligible studies published in Chinese. Some studies were only indexed in the CBM database but not indexed in the databases selected in the meta-analysis by Lao et al. [28] and Wang et al. [29], which could lead to location bias and might bias the effect estimate of a meta-analysis. Furthermore, a large number of new case–control studies have been published since 2008. Hence, to provide the most comprehensive assessment of the associations between the XRCC1 polymorphisms and bladder cancer risk, we performed an updated meta-analysis of all available studies. The meta-analysis was carried out by critically reviewing 24 individual case–control studies on the R399Q polymorphism, 15 studies on R194W polymorphism, and 7 studies on R280H polymorphism. Subgroup analyses were mainly done by ethnicity and by smoking status. Heterogeneity analysis and sensitivity analysis were also critically performed to ensure the epidemiological credibility of this meta-analysis. We found that the XRCC1 R399Q polymorphism was associated with a decreased bladder cancer risk among smokers (AA vs. GG: OR=0.693, 95%CI= 0.515-0.932, *P*=0.015 and recessive model AA vs. GA+GG: OR=0.680, 95%CI= 0.515-0.898, *P*=0.007, respectively). The R194W and R280H polymorphisms were both associated with increased bladder cancer risk among Asians (TT+CT vs. CC:OR = 1.327, 95% CI 1.086–1.622, *P*=0.006 for R194W, and AA+GA vs. GG: OR=2.094, 95% CI 1.211–3.621, *P*=0.008 for R280H, respectively).

The bladder, due to being the urine collecting area, is prone to contact with carcinogens. It is well established that the carcinogenesis of bladder cancer is a result of the interaction between environmental factors and genetic background. Besides the role of genetic variants, smoking behavior shows a major effect on the bladder cancer susceptibility [53]. It has been reported that smoking increased bladder cancer risk fourfold [54]. It is thought that smoking increased the risk due to chemicals such as hydrocarbons, arylamines, nitrosamines, and the formation of reactive oxygen species as by-products of the above compounds [55] that are known to induce bulky adducts, base damage, and DNA strand breaks in the bladder epithelium. DNA repair mechanisms are paramount in correcting the changes on DNA and provide unmutated DNA while replication goes on [56]. Therefore, constitutional variation in the ability to repair DNA base damage might lead to smoking-related cancers. This meta-analysis indicated that XRCC1 R399Q polymorphism was associated with decreased bladder cancer risk among smokers under the recessive genetic model and the homozygote contrast. The results were in accordance with previous published meta-analysis [28] and other epidemiological case-control studies [31].

When stratified by ethnicity, the R194W polymorphism was associated with increased bladder cancer risk among Asians (TT+CT vs. CC:OR = 1.327, 95% CI 1.086–1.622, *P*=0.006) but not among Caucasians and Africans, and it was the same with the R280H polymorphism (AA+GA vs. GG: OR=2.094, 95% CI 1.211–3.621, *P*=0.008 for R280H among Asians). These inconsistent data among the different ethnicities may indicate different effects of the XRCC1 R194W and R280H polymorphisms on bladder cancer risk in different ethnic genetic backgrounds. Nevertheless, owing to the limited number of relevant studies among Asians and Africans included in this meta-analysis, the observed ethnic difference in this meta-analysis is also likely to be caused by chance because studies with small sample sizes may have insufficient statistical power to detect a slight effect or may have generated a fluctuated risk estimate. Currently there are limited studies on XRCC1 R194W and R280H polymorphisms and bladder cancer risk among Asian populations and African populations. Therefore, large and carefully designed case–control studies need to be performed to provide the best evidence for the possible associations between the XRCC1 R194W and R280H polymorphisms and bladder cancer risk among Asian populations and African populations.

Heterogeneity is a potential problem when interpreting the results of all meta-analyses, and finding the sources of heterogeneity is one of the most important goals of meta-analysis [57]. In the present meta-analysis, we assessed the between-study heterogeneity by using three different methods including the chi-square based *Q* statistic test to test for heterogeneity, the *I* ^2^ statistic to quantify the between-study heterogeneity, and Galbraith plots to spot outliers as the possible major sources of heterogeneity. Meta-regression analysis was also applied to better investigate possible sources of heterogeneity that might influence results. Generally, there was significant between-study heterogeneity in all pooled analyses of total eligible studies for R399Q and R280H polymorphisms (*P*_*Q*_ values for R399Q, and R280H polymorphisms were all less than 0.10, or *I* ^2^ values were larger than 50.0%), which suggested obvious inconsistency of effects across those included studies. To find the major sources of heterogeneity, we first performed several subgroup meta-analyses by ethnicity, smoking status, and by studies in HWE. Subgroup analyses showed that the heterogeneity was still significant in Asians when stratified by ethnicity, while it was removed in the other subgroup analyses, indicating that heterogeneity might result from the inconsistency of effects across those studies included from Asian populations. For the R399Q polymorphism, Galbraith plots spotted 2 studies [44,50] as the outliers and the possible major sources of heterogeneity, while Galbraith plots for the R280H polymorphism spotted 1 study [50] as the outlier and the possible major source of heterogeneity. Interestingly, The studies spotted as the outliers and the possible major sources of heterogeneity were all from Asian populations, which further identified that the inconsistency of effects across those studies from the above population might be the major sources of heterogeneity in this meta-analysis, and the inconsistency of effects may be caused by the differences among those studies in the selection of controls, the population from different geographic regions, prevalence of life-style factors, or other unknown aspects. Meta-regression analysis in present meta-analysis further suggested that ethnicity might be important sources of between-study heterogeneity for the R399Q and R280H polymorphisms in subgroup analyses of Asians, which further validated the hypothesis above.

Some possible limitations in this meta-analysis should be acknowledged. Firstly, the eligibility criteria for inclusion of cases were different from each other. For instance, the cases in some studies were selected from transitional cell carcinoma [44,48], while the cases in other studies were selected from undefined bladder cancer individuals. As the studied genotypes may not only influence bladder carcinogenesis but also influence the histopathological type of bladder cancer, it would be better to investigate possible biases if more subgroup analyses were performed according to the different characteristics of case groups. However, we could not make it owing to the limited reported information in the included studies on such aspects and this point is a major limitation of this meta-analysis. Additionally, misclassification bias was possible. For example, most studies could not exclude latent cancer cases in the controls. Finally, gene–environment interactions were not fully addressed in this meta-analysis due to the lack of sufficient data As is generally accepted, aside from genetic factors, smoking status, and carcinogenic chemicals are major risk factors for bladder cancer; however, we could not perform subgroup analyses based on environmental exposure owing to the limited reported information on such associations in those included studies.

Despite these limitations, this meta-analysis suggests that XRCC1 R194W and R280H polymorphisms were both associated with increased bladder cancer risk among Asians. However, the XRCC1 R399Q polymorphism may play a protective role against bladder cancer among smokers. Further studies with larger sample sizes and rigorous design are still needed, especially for investigating the effects of the gene–gene and gene–environment interaction.

## Supporting Information

Figure S1 Flow diagram of included studies for this meta-analysis.
(TIF)

Table S1 **Checklist of items to include when reporting a systematic review or meta-analysis.**
(DOC)
