# Supplementary figures and images for: DNA Repair Gene XRCC1 Polymorphisms, Smoking, and Bladder Cancer Risk: A Meta-Analysis
Source: PLoS One. 2013 Sep 9;8(9):e73448. doi: 10.1371/journal.pone.0073448 (PMC3767803; doi:10.1371/journal.pone.0073448)

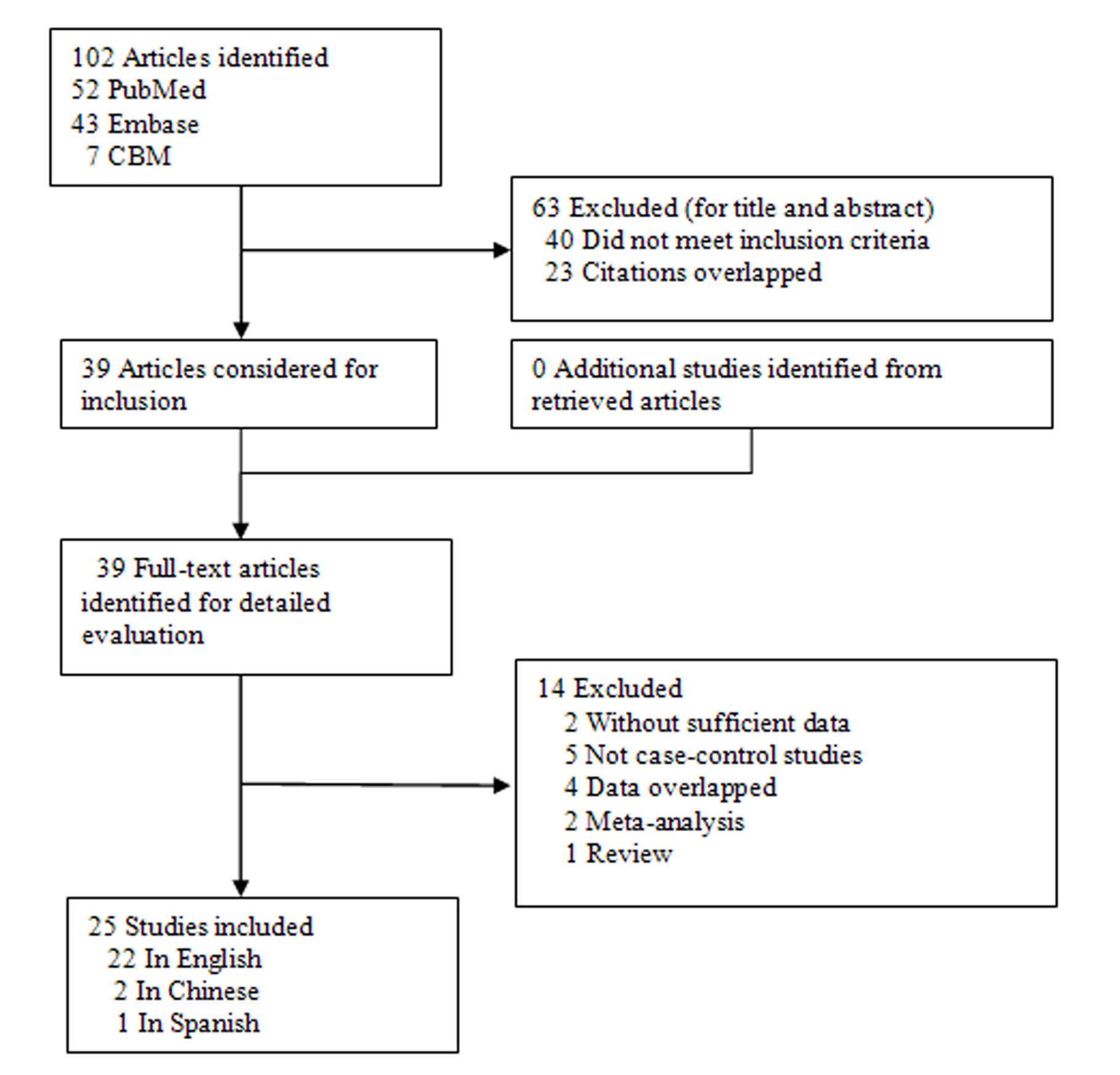

Supplement: Figure S1 — Flow diagram of included studies for this meta-analysis. (TIF) [file pone.0073448.s001.tif]
